# Uterine leiomyosarcoma with central nervous system metastases

**DOI:** 10.3332/ecancer.2015.515

**Published:** 2015-03-05

**Authors:** Carina Meira Abrahão, Fernando Cotait Maluf

**Affiliations:** Centro Oncológico Antônio Ermírio de Moraes, Rua Maestro Cardim, 769, Bela Vista, São Paulo, Cep 01323-001, Brazil

**Keywords:** leiomyosarcoma, metastasis, central nervous system

## Abstract

Leiomyosarcoma (LMS) is a rare tumour and comprises 2–3% of all malignant uterus neoplasms [1]. Leiomyosarcoma is characterised by aggressive behaviour, high recurrence rates, and poor overall survival, despite multimodal treatment [3]. Surgery is the main treatment and consists of total abdominal hysterectomy. A randomised trial consisting of 224 patients diagnosed with uterine sarcomas stage I and II showed that adjuvant radiotherapy improves locoregional control. The role of adjuvant chemotherapy is still unclear [1]. Unfortunately, roughly 50% of patients with organ-confined disease will usually develop distant metastasis to lung, peritoneum, liver, pelvic, and para-aortic lymph nodes. Brain metastases are extremely rare [5].

In this article we describe two patients with a diagnosis of leiomyosarcoma who developed metastasis to the central nervous system (CNS). A literature review was done regarding this unusual metastatic site of dissemination in such patients.

**CASE 1:** This is a case of a 45-year-old woman, without comorbidities, who had a history of vaginal bleeding. In February 2009 she underwent simple hysterectomy and pathology showed benign leiomyomas without atypia associated with chronic cervicitis. Seven months after surgery she developed abdominal pain. Computed tomography (CT) of the abdomen and pelvis revealed metastatic disease to the lungs with associated enteroenteric intussusception at the jejunum level. The patient underwent enterectomy with primary anastomosis and pathology revealed high-grade uterine leiomyosarcoma. Immunohistochemistry (IHC) was positive for smooth actin muscle and desmin. Ki67 was >25%. Thirty days after the surgery she developed left pleural effusion and underwent a left chest drainage. A postoperative CT of the chest and abdomen showed lung, bone, and liver metastases. The patient was started on intravenous (IV) gemcitabine at a dose of 900 mg/m^2^ on days one and eight and IV docetaxel on day eight at the dose of 100 mg/m^2^ every three weeks for a total of eight cycles. The patient achieved a partial response and no grade III or IV toxicities. Eight months later the disease had progressed into her lungs, and hence she was started on IV ifosfamide at the dose of 1800mg/m^2^ IV from days one to five in association with IV epirubicin at the dose of 60 mg/m^2^ on days one and two every three weeks for a total of six cycles. The patient achieved a partial response to the treatment and reasonable tolerance. In September 2011 she presented with left motor deficit and headaches. A brain CT showed two brain tumours on the parietooccipital region, right as well as the left. She underwent surgical resection of both lesions and pathology revealed metastatic leiomyosarcoma. Postoperative whole-brain radiotherapy was administered at a total dose of 40 Gy divided in 20 fractions. The patient has been followed since then with no further treatment. In March 2012 the disease progressed to her lung and bones. At that time she was treated with IV ifosfamide at a dose of 1350 mg/m^2^ IV from days one to five in association with IV liposomal doxorubicin at the dose of 35 mg/m^2^ on day one every three weeks for a total of six cycles with a minor response followed by maintenance anastrozole at the dose 1mg. Six months after the end of chemotherapy, she developed one new metastasis in the frontal left lobe. She underwent surgery followed by ifosfamide 1.5 mg/m^2^ for four days combined with Doxil 26 mg/m^2^ (25% dose reduction) every four weeks with good tolerance. In December 2013 the disease progressed to her lungs and then she was initiated on IV trabectedin at the dose of 1.2 mg/m^2^ every three weeks with disease control.

**CASE 2:** This is a case of a 51-year-old female, with a past history of type II diabetes mellitus and thalassemia minor. In April 2007 she had lower abdomen pain. Image studies including magnetic resonance image (MRI) of the pelvis revealed a large mass posterior to the uterus measuring 11 x 10 x 11.5 cm. She underwent simple hysterectomy and pathology revealed an intermediate grade leiomyosarcoma. Further staging with CT of the chest showed metastatic disease to the lung and in December 2007 she was started on IV gemcitabine at the dose of 700 mg/m^2^ on days one and eight and IV docetaxel on day eight at the dose of 70 mg/m^2^ every three weeks for a total of six cycles. The patient achieved partial response. During the period off chemotherapy she had progression of disease. She was then started on IV ifosfamide 1800 mg/m^2^ from days one to five plus IV epirubicin 60 mg/m^2^ on days one and two every three weeks for a total of six cycles, with good tolerance. The patient achieved a partial response.

In February 2009, she had tonic-clonic seizures. An MRI of the brain revealed an isolated mass in postcentral gyrus. She underwent surgical resection and pathology revealed metastatic leiomyosarcoma. At that time there was no evidence of progression at other sites. Six months later she had progression of disease in the lungs and in the surgical bed of the CNS. Treatment administered consisted of whole-brain radiation with 3750 cGy total dose followed by a boost in the surgical bed. Gemcitabine 700 mg/m^2^ and docetaxel 70 mg/m^2^ were initiated after radiation for a total of three cycles with treatment stopped because of progression of disease in the lungs. In June 2009, oral temozolomide at the dose of 150 mg/m^2^ from days one to six every 28 days was started for a total of two cycles with no response. The patient was then re-treated with epirubicin 60 mg/m^2^ plus ifosfamide 1800 mg/m^2^ for a total of five cycles with partial response. In April 2010 the patient developed a new cerebral metastasis at the left parietal area treated with radiosurgery. Two months later, the patient developed abdominal pain because of bowel perforation and died.

## Discussion

Uterine leiomyosarcoma is an uncommon malignant tumour of the uterus and is associated with a high risk of recurrence and death even at earlier stages [1]. Like we have seen in our patients that we have reported on, instances of misdiagnosis is common. In many patients, the initial hypothesis pre-operatively is that of a benign smooth muscle disease. We also see that in some cases even at the time of myomectomy or hysterectomy, pathology may mislead as a benign tumour. The cornerstone treatment consists of total hysterectomy.

Apparently, there is no clear benefit in performing salpingo-oophorectomy or lymph node dissection [3]. Adjuvant external beam radiation is recommended to stage I and II disease based on the results of a randomised trial including 224 patients that showed superior local control rates comparing to observation with no impact on progression-free survival (PFS) and overall survival (OS) [8]. The role of adjuvant chemotherapy is unclear and one randomised trial including doxorubicin alone has not demonstrated a survival benefit [9]. Unlike from carcinosarcoma, which usually metastasise to the lymph nodes and peritoneum, uterine leiomyosarcoma primarily presents with a hematogenous spread. Lung is the most common site of distant metastasis and because of that initial staging should include a chest-x-ray or a CT of the thorax. Interestingly, in various other solid tumour types such as germ cell tumour, gestational trophoblastic disease, rectal cancer [2], presence of lung metastasis is a risk factor to CNS involvement [2]. Our two patients had lung metastasis prior to central nervous system involvement.

Systemic therapies for uterine leiomyosarcomas have evolved during the last two decades. Asides from anthracyclines and ifosfamide, newer agents have shown activity in advanced disease such as taxanes, gemcitabine, and temozolomide [10]. A phase II trial evaluated the role of adjuvant chemotherapy which included 47 patients with uterine high-grade leiomyosarcoma. The treatment consisted of four cycles of gemcitabine plus docetaxel (standard doses) followed by a further four cycles of doxorubicin. The rates of PFS at two and three years were 78% and 57%, respectively [11].

Boyar *et al* have reported in a phase II trial including 25 patients with and without prior systemic therapy treated with temozolomide to have a response rate of 32%. This study showed that temozolomide was well tolerated and appears to be active even in pretreated patients [12]. In general, the appearance of metastasis to the CNS was extremely rare and was restricted to 14 cases as reported in the literature. One hypothesis to explain the possibility of CNS involvement from uterine leiomyosarcoma possibly becoming a more frequent finding is based on the fact that newer chemotherapy agents have been developed for this disease with possible better systemic control [7]. As a consequence disease progression to CNS may occur because of some limitations of chemotherapeutic agents in achieving adequate concentrations in part because of the brain-blood-barrier. Supporting this hypothesis, the two patients we have reported on had CNS involvement despite the fact that systemic disease was controlled. In general, CNS involvement is supratentorial, with the frontal and parietal lobes most affected [6]. The neurological deficits depend on the specific area affected.

Considering the limitations of that data available in patients with uterine leiomyosarcoma and CNS, it seems that surgical resection followed by whole brain irradiation represents a solid option in the management of this rare condition [7]. Also, stereotactic radiosurgery can also be considered [7]. The outcome of patients with brain metastasis is poor, with a median survival time of only four months [2].

New treatment strategies for soft tissue sarcoma are currently being studied. Pazopanib, a multitargeted tyrosine kinase inhibitor, has single-agent activity in patients with advanced non-adipocytic soft-tissue sarcoma. The Europena Organisation of Research and Treatment of Cancer (EORTC) phase III conducted a study with 372 patients with advanced soft tissue sarcoma, randomising them to pazopanib (800 mg/day) versus placebo. Pazopanib led to increased PFS (five versus two months hazard ratio (HR) = 0.31; 95% confidence interval (CI): 0.67 to 1.11), but no increase in OS (12.5 versus 10.7 months, HR 0.86, 95% CI 0.67-1.1) [13].

There are several case reports with a description of durable responses with the use of trabectedin in patients with uterine leiomyosarcoma pretreated. Trabectedin is a marine-derived antineoplastic compound isolated from the Caribbean tunicate Ecteinascidia turbinata, and it is currently produced synthetically. The efficacy of trabectedin 1.5 mg/m^2^ 24-hour intravenous (IV) infusion every three weeks (every three weeks 24-hour) in patients with heavily pretreated, advanced/metastatic soft tissue sarcomas (STS) was previously evaluated in a few nonrandomised phase II studies. A randomised clinical trial showed a statistically significant clinical benefit (26.6% reduction in the relative risk of progression, 61% increase in median time to progression) for patients treated with trabectedin [14]. Response rates have been highest in the myxoid/round cell liposarcoma and leiomyosarcoma subtypes [14].

Eribulin inhibits microtubules via a mechanism that is distinct from other microtubule-targeting agents such as taxanes. Modest efficacy in LMS and adipocytic sarcomas was suggested in a phase II trial in which 128 patients, with a variety of sarcoma histotypes, all received eribulin. The study achieved the primary endpoint with PFS at 12 weeks, but it deserves further study in this setting [15].

Those treatments may be considered as an option to control advanced sarcomas in patients after failure of available standard-of-care therapies. Since these drugs were not available at the time the patients were treated, they were not used in the above patients.

## Conclusion

The two cases we have reported on suggest that patients with metastatic uterine leiomyosarcoma, in particularly to lungs, who achieve systemic control and prolonged survival, are at risk of developing CNS involvement. In this regard, surgical resection of the metastatic disease, if feasible, followed by radiation therapy should be considered in order to achieve optimal CNS control.
